# Intermediate observers based robust fault estimation for multi-agent systems under communication constraints via switching scheme

**DOI:** 10.1098/rsos.230736

**Published:** 2023-12-13

**Authors:** Yue Quan, Ruopeng Sun, Shichuan Ding, Hai Guo, Fuzhi Hu, Di Zhang, Xiaoyu Fan

**Affiliations:** ^1^ Anhui Science and Technology University, Bengbu 233000, Anhui, People’s Republic of China; ^2^ Anhui University, Hefei 230601, Anhui, People’s Republic of China

**Keywords:** multi-agent system, communication constraints, variable sampling strategy, intermediate observer, fault estimation

## Abstract

This paper addresses the problem of robust fault estimation for multi-agent systems (MASs) under communication constraints. Taking into account the possible data packet loss (DPL) in the information interaction of each subsystem, MASs are remodelled as switching systems by introducing a variable sampling strategy. Then, using the local information among agents, a novel intermediate observer design method based on switching scheme is proposed to estimate faults of MASs. Combining Lyapunov’s criterion and linear matrix inequality, sufficient conditions for the intermediate observer to be exponentially stable and have *H*_∞_ performance against bounded disturbances and the DPL are given. Finally, some simulations are provided to verify the effectiveness of the proposed method.

## 1. Introduction

Multi-agent systems (MASs) are distributed systems composed of a limited number of subsystems with completely independent computing capability. Due to its excellent characteristics and wide application prospects in engineering, the theory of MASs has been widely studied in many aspects and has achieved fruitful research results [1,2]. At present, MAS theory has become an important tool to solve large-scale complex system problems, and has been widely used in many fields [3,4].

With the continuous expansion of the scale of systems, the relationship between the subsystems becomes more and more complex. Different from the traditional centralized system, MASs are typical distributed systems. Due to the limitation of the communication network and the computing ability of the agent subsystem itself, the agent subsystem only exchanges and calculates the information of neighbour agent, but cannot obtain the global information of the whole system. For MASs, this distributed network structure is a double-edged sword. On the one hand, it improves the system’s strong robustness and the emergence of swarm intelligence. On the other hand, the absence of the central entity node of the monitoring system’s global state also increases the probability of system failure. Moreover, the lack of central monitoring node may also lead to a single fault spreading to the whole system through the cooperation between nodes, which will seriously threaten the safe operation of MASs. Therefore, the research on effective fault detection (FD) algorithms for MASs and the improvement of system reliability is a key problem that urgently needs to be solved in the field of MASs and limits development of MASs.

According to the implementation methods, FD algorithms can be divided into data-driven FD and analytic model based FD. At present, the FD of MASs mainly improves the above two methods to meet the requirements of the distributed system structure of MASs. Dang *et al.* [5] and Peng *et al.* [6] studied the problem of FD of data-driven MASs. Using the method of mathematical statistics, it analysed the historical data of the system, extracted the fault characteristics and trained the data of each node and finally realized the FD of the system. This method requires a large amount of historical data to train each node. It increases the computational burden of the system. On the other hand, the limitations of historical data cannot fully meet the dynamic characteristics and real-time requirements of MASs.

Different from the data based multi-agent FD method, the analytic model based FD does not require a lot of data training and it does not increase the computational burden of the system. Hence, the analytic model based FD can better meet the real-time requirements of the system and adapt to the dynamic characteristics of the MAS. Therefore, FD methods based on analytical models have been widely studied and applied in MASs. Among them, FD of distributed systems with unknown input observers (UIOs) is an effective method in this field, and fruitful research results have been achieved in FD of MASs. Shames *et al.* [7] proposed a FD framework based on UIOs for MASs, gave sufficient conditions for the existence of UIOs in an ideal topology network structure, and realized online FD of MASs. Considering the influence of uncertain factors in the MAS network, a series of improvements of results of Shames *et al.* have been researched. Liu *et al.* [8] proposed a FD method for UIOs of high-order MASs. By using UIOs, Chen *et al.* [9] studied the problem of FD for MASs with nonlinear disturbances, and Liang *et al.* [10] and Zhou *et al.* [11] studied the problem of FD for MASs with network delays. In addition, other aspects of FD of MASs based on UIOs are considered, such as FD of MASs in switching topology, FD of MASs in discrete systems and FD of MASs in finite time. Although many achievements have been made in FD of MASs based on UIOs, there are still some shortcomings. Firstly, the existence of UIOs requires strict matching conditions, which requires that the system parameter matrix must meet strict matrix rank conditions, increasing the difficulty of determining the unknown parameters of the system and reducing the flexibility of design. Secondly, the FD method of MAS based on UIO can only detect a single fault of the system effectively, but cannot detect multiple faults. Thirdly, the FD method of UIO can carry out fault alarm, but it cannot effectively estimate fault signals. These shortcomings limit the application of UIO in MAS FD.

Comparing with the above research results, fault estimation (FE) is a more effective method to achieve FD. FE can not only alert the fault signal, but also effectively estimate the signal state, which is helpful for further processing of system faults. Intermediate observer is an effective method for distributed fault estimation (DFE) [12]. Zhu *et al.* [13,14] proposed a FE method based on distributed intermediate observer, which can realize FD of MASs with undirected topology network. Considering that in the actual system, the node information in the MAS may be transmitted unidirectionally, Han *et al.* [15] and Liu *et al.* [16] proposed a FE method for the distributed intermediate observer of the MASs with a directed network topology.

In addition to being affected by unknown disturbance, communication delay, measurement noise and communication topology, MASs may also be affected by more complex factors, such as data packet loss (DPL), time-varying sampling period, etc. The emergence of these complex factors may make the MAS more complex. At present, the problem of MAS control under the influence of DPL has gradually attracted people’s attention [17,18]. However, in the aspect of FD of MASs, research on FD of DPL is relatively less. The objective existence of DPL may aggravate the vibration of MASs, thus increasing the difficulty of FD. Li *et al.* [19] proposed a FD method for MASs with DPL in the sensor controller link. In practice, DPL may occur in the sensor controller link, the controller executive link, or even both links. Zhang & Zhang [20] proposed a FD method for networked control systems with time-varying sampling and uncertain DPL rate by using *δ* operator method for MASs with simultaneous DPL from sensor to controller and controller to executive link. It should be pointed out that although these two methods have realized the FD of MASs with DPL, they have not realized the effective estimation of the fault signal and cannot provide a reference for the identification of future fault types. As far as we know, there are no published research results on the problem of FE of MASs with DPL. Therefore, it will be more meaningful and challenging to study the problem of FE of MASs under the influence of DPL.

Based on the analysis of the above problems, this paper studies the FE problem of MASs with DPL by using the intermediate observer method. Firstly, the problem of DPL in the data transmission link is transformed into the problem of time-varying sampling period control of the system by using the input holding strategy of the controller. Furthermore, using Delta operator method, the MASs with DPL are represented as a switching system with time-varying sampling period. Secondly, using the relative state information of the MASs, an intermediate observer satisfying the switching system model is constructed to realize the FE of MASs with DPL. Finally, combined with the mean dwell time and Lyapunov stability criterion, the conditions of exponential stability for the intermediate observations designed in this paper are given.

Compared with the existing achievements on multi-agent FD or FE, the main contributions of this paper can be summarized as follows:

1. The system model considers the simultaneous DPL of the sensor controller link and the controller executive link. Compared with the fault model in [12–16], the model in this paper is more consistent with the requirements of the actual system. Furthermore, the MAS with DPL is transformed into a switched system by using the method of time-varying sampling period, where the small network delay can be ignored when the network delay is smaller than the sampling period.
2. Using the relative state information of MASs, an intermediate observer with switched system modes is constructed. Compared with the UIO in [8–11], this observer does not depend on the matching relationship of the parameter matrix in the system, and has strong design flexibility. Compared with the results in [15,16], this observer has better robustness to DPL. Compared with [20], the observer designed in this paper can not only realize the faulty alarm for multi-faults, but also effectively estimate the fault state signal, which can pave the way for further fault processing.
3. Using the concept of average dwell time (ADT), the relationship between the stability of the intermediate observer of the switched system, the DPL rate and the number of consecutive packets is given under the condition of exponential stability. By using Lyapunov stability criterion, the matrix inequality (LMI) satisfied by the undetermined parameters of the intermediate observer is obtained. The dimension of the LMI is only related to the state dimension of a single agent, but not to state dimension of the entire system. Compared with the dimension of LMI in [12–16], the dimension of LMI in this paper is less, which can effectively reduce the computational burden of the system.

## 2. Problem description

In MASs, each subsystem is composed of independent sensors, controllers, actuators, network data transmitters and receivers. These subsystems realize information interaction through the network and form a complete network distributed system. Each agent interacts with each other through the network. The topological network of MASs can be denoted as G=(V,ε,A), where *V* = {*V*_*i*_|*i* = 1, 2, …, *N*} represents the node set, *V*_*i*_ represents the *i*th node, *A* = [*a*_*ij*_] ∈ *R*^*N*×*N*^ is the adjacency matrix of *G* and *N* is the number of agents in MASs. *a*_*ij*_ is determined by the edge set ε of graph *G*. For example, *a*_*ij*_ = 1 if $(V_{i},V_{j})\in \varepsilon$ when *i* ≠ *j*, otherwise *a*_*ij*_ = 0. In the undirected topology, $(V_{i},V_{j})\in \varepsilon$ and $(V_{j},V_{i})\in \varepsilon$ exist at the same time, that is, *a*_*ij*_ = *a*_*ji*_. $L=[l_{ij}]\in {ℜ}^{N\times N}$ is the Laplacian matrix of *G*, where $l_{ij}=\sum_{\;j=1}^{N}a_{ij}$ when *i* = *j* and *l*_*ij*_ = −*a*_*ij*_ when *i* ≠ *j*.

The dynamic model of the *i*th agent subsystem is considered as follows:

2.1 $$\left\{ \begin{array}{c} {\dot{x}}_{i}(t)=A_{0}x_{i}(t)+B_{0}u_{i}(t)\;\\ y_{i}(t)=Cx_{i}(t)\; \end{array}\right.,\;i=1,2\ldots ,N,$$

where $x_{i}(t)\in {ℜ}^{n},u_{i}(t)\in {ℜ}^{n_{u}},y_{i}(t)\in {ℜ}^{n_{y}}$, respectively, represent the state, control input and state output of the *i*th agent subsystem, *A*_0_, *B*_0_, *C* are known real matrices with appropriate dimensions, satisfying that (*A*_0_, *B*_0_) is controllable and (*A*_0_, *C*) is measurable.

**Figure 1.**
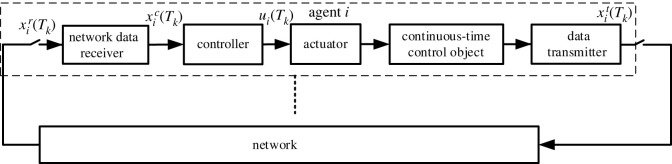
Structure of MASs with DPL.

It is assumed that the sensor is time-driven and samples the system with a fixed sampling period. Let *T*_*k*_ (*k* = 0, 1, 2, …) represent the sampling time of the system and $h\overset{\mathrm{def}}{=}T_{k}-T_{k-1}$ represent the sampling period. The structure of MASs with time-driven sensors can be shown as in figure 1. Where $x_{i}^{t}(T_{k})$ represents the information sending to network of agent *i*, $x_{i}^{r}(T_{k})$ represents the information receiving from network of agent *i*, $x_{i}^{c}(T_{k})$ and *u*_*i*_(*T*_*k*_), respectively, represent the input information and output information of the controller in agent *i*.

**Figure 2.**
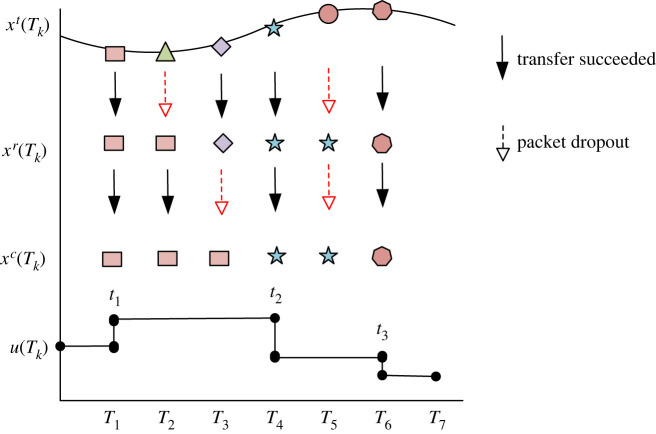
Control sequence of MASs with DPL.

Due to the limitation of network bandwidth, DPL may collide or communication nodes may fail to complete in the process of network transmission, resulting in the loss of packets to be transmitted. DPL may occur on the agent’s network data sending link or network data receiving link, or on two links at the same time. In order to reduce the impact of DPL on MASs, both the network data receiver and controller adopt the event driven control mode. If DPL occurs during data transmission, they will keep the last data, i.e. $x_{i}^{r}(t_{k})=x_{i}^{r}(t_{k-1})$, *u*_*i*_(*t*_*k*_) = *u*_*i*_(*t*_*k*−1_) when there is DPL at *T*_*k*_. The impact of DPL on system dynamics is described in figure 2.

At *T*_1_, all data are successfully transmitted, and the output signal of the implementation controller is *u*(*T*_1_). At *T*_2_ and *T*_3_, DPL occurs, respectively, in data transmission and data reception. The data receiver will keep the data of the previous time, and the controller will also keep the output of the previous time, that is *u*(*T*_2_) = *u*(*T*_1_), *u*(*T*_3_) = *u*(*T*_1_). At *T*_4_, the data are successfully transmitted, and the output signal of the controller is *u*(*T*_4_). At *T*_5_, DPL occurs simultaneously in the sending process and the receiving process. At this time, the controller will maintain the original output, *u*(*T*_5_) = *u*(*T*_4_). According to the output characteristics of control signals, the MAS with DPL can be regarded as a sampled control system with variable sampling period.

**Assumption 2.1.**

The network induced delay is ignored, and the number of continuous DPL in the network is bounded and supposed *d*.

Let *h*_*k*_ = *t*_*k*+1_ − *t*_*k*_ denote the variable sampling period, and *t*_*k*_ denotes the sampling time. Based on the results of the analysis of figure 2, it can be got that *h*_*k*_ = *n*_*k*_ *h*. According the assumption 1, it is defined *h*_*k*_ ∈ {*h*, …, (*d* + 1)*h*}, $n_{k}\in Z_{\delta }\overset{\mathrm{def}}{=}\{1,\ldots ,d+1\}$. If the number of DPL in MASs is bounded and *d*, then the system (2.1) may have the following description of the control system model with non-equidistant sampling periods:

2.2 $$\left\{ \begin{array}{c} {\dot{x}}_{i}(t)=A_{0}x_{i}(t)+B_{0}u_{i}(t_{k})\;\\ y_{i}(t)=Cx_{i}(t)\; \end{array}\right.,\;\forall t\in [t_{k},t_{k-1}),i=1,2,\ldots ,N.$$

By discretizing the system (2.2) with sampling period *h*_*k*_, the following discrete-time system model (2.3) can be obtained:

2.3 $$\begin{array}{cc} & x_{i}(t_{k+1})=A(h_{k})x_{i}(t_{k})+B(h_{k})u_{i}(t_{k})\\ \mathrm{and}\;\;\;\;\; & y_{i}(t_{k})=Cx_{i}(t_{k}),\;\; \end{array}\}\;$$

where $A(h_{k})=e^{A_{0}h_{k}}$, $B(h_{k})=\int_{0}^{h_{k}}e^{A_{0}\tau }d\tau B$. Let $A_{1}=e^{A_{0}h}$ and $B_{1}=\int_{0}^{h}e^{A_{0}\tau }d\tau B$, then *A*(*h*_*k*_) and *B*(*h*_*k*_) can be described as

2.4 $$A(h_{k})=e^{A_{0}h_{k}}=e^{A_{0}n_{k}h}=A_{1}^{n_{k}}$$

and

2.5 $$\begin{array}{rl} B(h_{k}) & =\int_{0}^{h_{k}}\;e^{A_{0}\tau }\;d\tau B\\ & =\left(\sum_{i=0}^{n_{k}-1}\int_{ih}^{(i+1)h}\;e^{A_{0}\tau }\;d\tau \right)B\\ & =\left(\sum_{i=0}^{n_{k}-1}{\left(e^{A_{0}h}\right)}^{i}\right)\int_{0}^{h}\;e^{A_{0}\tau }\;d\tau B\\ & =\left(\sum_{i=0}^{n_{k}-1}A_{1}^{i}\right)B_{1}. \end{array}$$

According to (2.4) and (2.5) *A*(*h*_*k*_) and *B*(*h*_*k*_) both depend on *n*_*k*_, while *n*_*k*_ can have at most *d* + 1 values. Furthermore, a piecewise continuous switching system signal $\delta (t_{k})\in Z_{\sigma }$ is introduced, and let $A_{\delta (t_{k})}=A_{1}^{\delta (t_{k})}$, $B_{\delta (t_{k})}=(\sum_{\;j=1}^{\delta (t_{k})}A_{1}^{\;j-1})B_{1}$, then system (2.3) is equivalent to the following form of switching system:

2.6 $$s_{\delta (t_{k})}^{i}\;:\;\left\{ \begin{array}{c} x_{i}(t_{k+1})=A_{\delta (t_{k})}x_{i}(t_{k})+B_{\delta (t_{k})}u_{i}(t_{k})\;\\ y_{i}(t_{k})=Cx_{i}(t_{k}),\; \end{array}\right.\;$$

where *δ*(*t*_*k*_) = *n*_*k*_ ∈ {1, 2, …, *d* + 1} represents a subsystem with continuous DPL of *n*_*k*_ − 1.

When MASs are affected by faults or external disturbance signals, the fault signals and external disturbance signals can be taken as the superposition signals of the system. Let $\;f_{i}(t_{k})\in {ℜ}^{n_{f}}$ and $d_{i}(t_{k})\in {ℜ}^{n_{d}}$, respectively, represent the fault signal vector and external disturbance of agent *i*. It is assumed that *f*_*i*_(*t*_*k*_) and *d*_*i*_(*t*_*k*_) are bounded and satisfy *f*_*i*_(*t*_*k*_) ∈ *l*_2_(0, ∞), *d*_*i*_(*t*_*k*_) ∈ *l*_2_(0, ∞). Then the dynamics of agent *i* affected by DPL, disturbance and faulty can be expressed as

2.7 $$s_{\delta (t_{k})}^{i}\;:\;\left\{ \begin{array}{c} x_{i}(t_{k+1})=A_{\delta (t_{k})}x_{i}(t_{k})+B_{\delta (t_{k})}u_{i}(t_{k})+F\;f_{i}(t_{k})+Ed_{i}(t_{k})\;\\ y_{i}(t_{k})=Cx_{i}(t_{k}),\; \end{array}\right.\;$$

where *F* and *E* are known real matrices with appropriate dimensions. To simplify the analysis difficulty of the problem, the following assumption is given.

**Assumption 2.2.**

The fault signal will only affect the output of the system and will not change the dynamic parameters of the MASs.

## 3. Main results

### 3.1. Intermediate observer

The intermediate variable about agent *i* is firstly construct to estimate the fault signal:

3.1 $$\phi_{i}(t_{k})=\;f_{i}(t_{k})-K_{1}x_{i}(t_{k}),$$

where *K*_1_ is an undetermined matrix. Then it can be got that *f*_*i*_ (*t*_*k*_) = *ϕ*_*i*_ (*t*_*k*_) + *K*_1_*x*_*i*_ (*t*). Combined with equation (2.7), it can be further obtained from equation (3.1):

3.2 $$\begin{array}{rl} \phi_{i}(t_{k+1}) & =\;f_{i}(t_{k+1})-K_{1}x_{i}(t_{k+1})\\ & =\;f_{i}(t_{k+1})-K_{1}(A_{\delta (t_{k})}x_{i}(t_{k})+B_{\delta (t_{k})}u_{i}(t_{k})\\ & \;+F\;f_{i}(t_{k})+Ed_{i}(t_{k}))\\ & =\;f_{i}(t_{k+1})-(K_{1}A_{\delta (t_{k})}+K_{1}FK_{1})x_{i}(t_{k})\\ & \;-K_{1}B_{\delta (t_{k})}u_{i}(t_{k})-K_{1}F\phi_{i}(t_{k})-K_{1}Ed_{i}(t_{k}). \end{array}$$

The following state observers are designed as

$$\begin{array}{rl} & {\hat{x}}_{i}(t_{k+1})=A_{\delta (t_{k})}{\hat{x}}_{i}(t_{k})+B_{\delta (t_{k})}u_{i}(t_{k})+F{\hat{f}}_{i}(t_{k})+\rho K_{2}\varsigma_{i}(t_{k}),\\ & {\hat{\phi }}_{i}(t_{k+1})=-K_{1}F{\hat{\phi }}_{i}(t_{k+1})-(K_{1}A_{\delta (t_{k})}+K_{1}FK_{1}){\hat{x}}_{i}(t_{k})\\ & \;\;\;-K_{1}B_{\delta (t_{k})}u_{i}(t_{k})+\rho K_{3}\varsigma_{i}(t_{k}),\\ & {\hat{f}}_{i}(t_{k})={\hat{\phi }}_{i}(t_{k})+K_{1}{\hat{x}}_{i}(t)\\ \mathrm{and}\;\;\;\; & {\hat{y}}_{i}(t_{k})=C{\hat{x}}_{i}(t_{k}), \end{array}$$

where ${\hat{x}}_{i}(t_{k})$, ${\hat{\phi }}_{i}(t_{k})$ and ${\hat{f}}_{i}(t_{k})$ represent the system state estimation, intermediate variable estimation and fault signal estimation at *t*_*k*_, respectively. $\varsigma_{i}(t_{k})=\sum_{\;j=1}^{N}a_{ij}[(y_{i}(t_{k}))-{\hat{y}}_{i}(t_{k}))-(y_{j}(t_{k}))-{\hat{y}}_{j}(t_{k}))]$ represents the system distributed output estimation error. *ρ* > 0, *K*_1_ and *K*_2_ are the undetermined gain matrices of the observer.

Let $e_{x}^{i}(t_{k})=x_{i}(t_{k})-{\hat{x}}_{i}(t_{k})$, $e_{\phi }^{i}(t_{k})=\phi_{i}(t_{k})-{\hat{\phi }}_{i}(t_{k})$ and $e_{f}^{i}(t_{k})=\;f_{i}(t_{k})-{\hat{f}}_{i}(t_{k})$ represent the state estimation error of the system, respectively. It can be further obtained that

3.3 $$\begin{array}{rl} e_{x}^{i}(t_{k+1}) & =x_{i}(t_{k+1})-{\hat{x}}_{i}(t_{k+1})\\ & =A_{\delta (t_{k})}x_{i}(t_{k})+B_{\delta (t_{k})}u_{i}(t_{k})+F\;f_{i}(t_{k})+Ed_{i}(t_{k})\\ & \;-A_{\delta (t_{k})}{\hat{x}}_{i}(t_{k})-B_{\delta (t_{k})}u_{i}(t_{k})-F{\hat{f}}_{i}(t_{k})-\rho K_{2}\varsigma_{i}(t_{k})\\ & =A_{\delta (t_{k})}e_{x}^{i}(t_{k})+Fe_{f}^{i}(t_{k})-\rho K_{2}\varsigma_{i}(t_{k})+Ed_{i}(t_{k}) \end{array}$$

and

3.4 $$\begin{array}{rl} e_{\phi }^{i}(t_{k+1}) & =\phi_{i}(t_{k+1})-{\hat{\phi }}_{i}(t_{k+1})\\ & =\;f_{i}(t_{k+1})-(K_{1}A_{\delta (t_{k})}+K_{1}FK_{1})x_{i}(t_{k})-K_{1}B_{\delta (t_{k})}u_{i}(t_{k})\\ & \;-K_{1}F\phi_{i}(t_{k})-K_{1}Ed_{i}(t_{k})+K_{1}F{\hat{\phi }}_{i}(t_{k+1})+(K_{1}A_{\delta (t_{k})}\\ & \;+K_{1}FK_{1}){\hat{x}}_{i}(t_{k})+K_{1}B_{\delta (t_{k})}u_{i}(t_{k})-\rho K_{2}\varsigma_{i}(t_{k})\\ & =-K_{1}Fe_{\phi }^{i}(t_{k})-(K_{1}A_{\delta (t_{k})}+K_{1}FK_{1})e_{x}^{i}(t_{k})-K_{1}Ed_{i}(t_{k})\\ & \;+\;f_{i}(t_{k+1})-\rho K_{3}\varsigma_{i}(t_{k}). \end{array}$$

It is noted that

3.5 $$\begin{array}{rl} e_{f}^{i}(t_{k}) & =\;f_{i}(t_{k})-{\hat{f}}_{i}(t_{k})\\ & =\phi_{i}(t_{k})+K_{1}x_{i}(t)-{\hat{\phi }}_{i}(t_{k})-K_{1}{\hat{x}}_{i}(t)\\ & =e_{\phi }^{i}(t_{k})+K_{1}e_{x}^{i}(t_{k}). \end{array}$$

Combining with (3.5), equation (3.3) can be rewritten as

3.6 $$e_{x}^{i}(t_{k+1})=(A_{\delta (t_{k})}+FK_{1})e_{x}^{i}(t_{k})+Fe_{\phi }^{i}(t_{k})-\rho K_{2}\varsigma_{i}(t_{k})+Ed_{i}(t_{k}).$$

Let $K_{1}=\varepsilon F^{T}$, where *F*^*T*^ is transpose matrix of *F* and ε>0 is an undetermined constant. Combining (3.4) and (3.6), it can be got the error dynamics about agent *i*:

3.7 $$\begin{array}{rl} & e^{i}(t_{k+1})={\bar{A}}_{1}\;e^{i}(t_{k})+E_{1}\omega_{i}(t_{k})-\rho {\bar{K}}_{2}\sum_{\;j=1}^{N}a_{ij}[(y_{i}(t_{k})-{\hat{y}}_{i}(t_{k}))-(y_{j}(t_{k})-{\hat{y}}_{j}(t_{k}))]\\ \mathrm{and}\;\; & Y_{\;}^{i}(t_{k})=\bar{C}\;e^{i}(t_{k}), \end{array}\}\;$$

where ${\bar{A}}_{1}=\left[\begin{array}{c} A_{\sigma (t_{k})}+\varepsilon FF^{T}\;F\\ -\varepsilon F^{T}(A_{\sigma (t_{k})}+\varepsilon FF^{T})\;-\varepsilon F^{T}F \end{array}\right]$, $E_{1}=\left[\begin{array}{c} E\;O\\ -\varepsilon F^{T}E\;I \end{array}\right]$, ${\bar{K}}_{2}=\left[\begin{array}{c} K_{2}\\ K_{3} \end{array}\right]$, $\bar{C}=\left[\begin{array}{c} I_{n}\;O\\ K_{1}\;I_{nf} \end{array}\right]$ and $\omega_{i}(t_{k})=\left[\begin{array}{c} d_{i}(t_{k})\\ f_{i}(t_{k+1}) \end{array}\right]$ can be regarded as an interference term in error dynamics (3.7), which is bounded and satisfies *ω*(*t*_*k*_) ∈ *l*_2_[0, ∞). Let *e*(*t*_*k*_) = [(*e*^1^(*t*_*k*_))^*T*^, {(*e*^2^(*t*_*k*_))^*T*^, …, (*e*^*N*^(*t*_*k*_))^*T*^]^*T*^, *ω*(*t*_*k*_) = [(*ω*^1^(*t*_*k*_))^*T*^, {(*ω*^2^(*t*_*k*_))^*T*^, …, (*ω*^*N*^(*t*_*k*_))^*T*^]^*T*^, and *Y*(*t*_*k*_) = [(*Y*^1^(*t*_*k*_))^*T*^, {(*Y*^2^(*t*_*k*_))^*T*^, …, (*Y*^*N*^(*t*_*k*_))^*T*^]^*T*^, then the error dynamic equation of the whole MASs can be obtained:

3.8 $$S_{\delta (t_{k})}\;:\;\left\{ \begin{array}{c} e(t_{k+1})=I_{N}⊗{\bar{A}}_{1}e(t_{k})-(\rho L⊗{\bar{K}}_{2}C)e(t_{k})+I_{N}⊗E_{1}\omega (t_{k})\;\\ Y_{\;}^{i}(t_{k})=I_{N}⊗\bar{C}e(t_{k}).\; \end{array}\right.$$

Obviously, the observer described above can effectively carry out the estimation of the state of the whole MASs when system (3.8) is convergent. Therefore, the observer design problem can be transformed into the convergence analysis of the system (3.8).

### 3.2. Stability analysis of error dynamics

Before the analysis, some definitions are given.

**Definition 3.1.**

[21] For the given switching signal *δ*(*t*_*k*_) and any *t*_*k*_ > *t*_0_ > 0, let *N*_*δ*_[*t*_0_, *t*_*k*_) represent the switching times of the switching signal *δ*(*t*_*k*_) at the interval [*t*_0_, *t*_*k*_). If there are *N*_0_ ≥ 0 and *τ*_*a*_ > 0 to satisfy *N*_*δ*_[*t*_0_, *t*_*k*_) ≤ *N*_0_ + ((*t*_*k*_ − *t*_0_)/*τ*_*a*_), then *τ*_*a*_ is called the ADT of the switching signal *δ*(*t*_*k*_). In this paper, *N*_0_ = 0.

**Definition 3.2.**

[22] Considering the system (3.8) with *ω*(*t*_*k*_) = 0, if there are normal values ς and *λ* < 1, $∥e(t_{k})∥\leq \varsigma \lambda^{t_{k}-t_{0}}∥e(t_{0})∥$ for any initiation condition $e(t_{0})\in {ℜ}^{n}$, then the system (3.8) is exponentially stable with exponential decay rate *λ*.

**Definition 3.3.**

[22] For any given *γ* > 0, the system (3.8) satisfies the following conditions:

(1) When *ω*(*t*_*k*_) = 0, system (3.8) is exponentially stable.
(2) Under zero initial condition, for any non-zero signal *ω*(*t*_*k*_), system (3.8) satisfies that $\sum_{k=0}^{\infty }e^{T}(t_{k})e(t_{k})<\sum_{k=0}^{\infty }\gamma^{2}\omega^{T}(t_{k})\omega (t_{k})$, then it is said that the system (3.8) has the *H*_∞_ performance, where *γ* is the *H*_∞_ performance level.

It is assumed that the system (3.8) resides in the subsystem $S_{\delta (t_{0})}$ ($\delta (t_{0})\in Z_{\delta }$) at the initial time *t*_0_. Let $t_{k_{1}},t_{k_{2}},\ldots t_{k_{d}}$ indicate the switching time in the [*t*_0_, *t*_*k*_), which satisfies $t_{0}<t_{k_{1}}<t_{k_{2}}<\cdots <t_{k_{d}}<t_{k}$, (*d* > 1). $S_{\delta (t_{k})}$ is switched by the sequence $\Pi \;:\;\{(\chi_{0},t_{0}),(\chi_{1},t_{k_{1}}),\ldots ,(\chi_{\varphi },t_{k_{\varphi }})|\chi_{\varphi }\in Z_{\delta },\varphi =1,\ldots ,d+1\}$. For the convenience of analysis, let $e_{\chi_{\varphi }}(t_{k})$ represent the output of *χ*_φ_th subsystems. Considering the state of the subsystem remains unchanged at the switching instant, we can get (3.9) when subsystem is switched from *χ*_φ−1_ to *χ*_φ_ at $t_{k_{\varphi }}:$

3.9 $$e_{\chi_{\varphi }}(t_{k_{\varphi }})=e_{\chi_{\left(\varphi -1\right)}}(t_{k_{\varphi }}).$$

Let $\gamma_{\chi_{\varphi }}$ denote the occurrence rate of DPL process of subsystem $S_{\chi_{\varphi }}(\chi_{\varphi }\in Z_{\delta })$, then it can be got that $\sum_{\varphi =1}^{d+1}\gamma_{\chi_{\varphi }}=1$ and $\gamma_{\chi_{\varphi }}\geq 0$. Let $n_{\chi_{\varphi }}$ represent the occurrence number of DPL of subsystem $S_{\chi_{\varphi }}$ at intervals [*t*_0_, *t*_*k*_). According to the definition of ADT, it can be got that $n_{\chi_{\varphi }}=\gamma_{\chi_{\varphi }}N_{\delta }[t_{0},t_{k})=\gamma_{\chi_{\varphi }}((t_{k}-t_{0})/\tau_{\alpha })$, and $\sum_{\varphi =1}^{d+1}n_{\chi_{\varphi }}=\sum_{\varphi =1}^{d+1}\gamma_{\chi_{\varphi }}((t_{k}-t_{0})/\tau_{\alpha })=(t_{k}-t_{0})/\tau_{\alpha }$. The subsystem $S_{\chi_{\varphi }}$ (φ = 2, 3, …, *d* + 1) is activated only when DPL occurs. Hence, DPL rate of the system can be described as

3.10 $$\alpha =\sum_{\varphi =2}^{d+1}\gamma_{\chi_{\varphi }}.$$

**Theorem 3.4.**

*For MASs with undirected connected topology, system* (3.8) *with* *ω*(*t*_*k*_) = 0 *is exponentially stable and satisfies the exponential decay rate* $\rho (\tau_{\alpha },\lambda )=\mu^{-(1/2\tau_{\alpha })}\lambda$ *if there are scalar* *λ* > 1, *μ* > 1, $\lambda_{\chi_{\varphi }}>1$ ($\chi_{\varphi }\in Z_{\delta },\varphi =1,\ldots ,d+1$), *positive definite matrix* $P_{\delta (t_{k})}$, $P_{\alpha }$ *and* $P_{\beta }$ ($\alpha ,\beta \in Z_{\delta }$) *to satisfy the following inequality:*

3.11 $$\Xi_{i}=\left[\begin{array}{cc} -P_{\delta (t_{k})} & P_{\delta (t_{k})}{\bar{A}}_{1}-\rho \psi_{i}P_{\delta (t_{k})}{\bar{K}}_{2}C)\\ {}* & -\lambda_{\delta (t_{k})}^{-2(t_{k+1}-t_{k})}P_{\delta (t_{k})} \end{array}\right]<0,\;i=1,2,\ldots ,N,$$

3.12 $$P_{\alpha }<\mu P_{\beta },(\alpha ,\beta \in Z_{\delta }),$$

3.13 $$\prod_{\varphi =1}^{d+1}\lambda_{\chi_{\varphi }}^{\gamma_{\chi_{\varphi }}}>\lambda >1,$$

*and*

3.14 $$\tau_{a}>\frac{\ln\mu }{2\ln\lambda }.$$

**Proof.**

Choosing a candidate Lyapunov function as

3.15 $$V_{\delta (t_{k})}(t_{k})=e_{\;}^{T}(t_{k})(I_{N}⊗P_{\delta (t_{k})})e(t_{k}),$$

when *ω*(*t*_*k*_) = 0, by (3.8) and (3.15), it follows that

3.16 $$\begin{array}{rl} & V_{\delta (t_{k})}(t_{k+1})-\lambda_{\delta (t_{k})}^{-2(t_{k+1}-t_{k})}V_{\delta (t_{k})}(t_{k})\\ & \;=e_{\;}^{T}(t_{k+1})P_{\delta (t_{k})}e(t_{k+1})-\lambda_{\;}^{-2(t_{k+1}-t_{k})}e_{\;}^{T}(t_{k})P_{\delta (t_{k})}e_{\;}(t_{k})\\ & \;=e_{\;}^{T}(t_{k}){\left(I_{N}⊗{\bar{A}}_{1}-(\rho L⊗{\bar{K}}_{2}C)\right)}^{T}(I_{N}⊗P_{\delta (t_{k})})(I_{N}⊗{\bar{A}}_{1}-(\rho L⊗{\bar{K}}_{2}C))e(t_{k})\\ & \;-\lambda_{\delta (t_{k})}^{-2(t_{k+1}-t_{k})}e_{\;}^{T}(t_{k})(I_{N}⊗P_{\delta (t_{k})})e(t_{k})\\ & \;=e_{\;}^{T}(t_{k})[{\left(I_{N}⊗{\bar{A}}_{1}-(\rho L⊗{\bar{K}}_{2}C)\right)}^{T}(I_{N}⊗P_{\delta (t_{k})})(I_{N}⊗{\bar{A}}_{1}-(\rho L⊗{\bar{K}}_{2}C))\\ & \;-\lambda_{\delta (t_{k})}^{-2(t_{k+1}-t_{k})}(I_{N}⊗P_{\delta (t_{k})})]e(t_{k}). \end{array}$$

If the following equation (3.17) holds:

3.17 $$\begin{array}{rl} & {\left(I_{N}⊗{\bar{A}}_{1}-(\rho L⊗{\bar{K}}_{2}C)\right)}^{T}(I_{N}⊗P_{\delta (t_{k})})(I_{N}⊗{\bar{A}}_{1}-(\rho L⊗{\bar{K}}_{2}C))\\ & \;-\lambda_{\delta (t_{k})}^{-2(t_{k+1}-t_{k})}(I_{N}⊗P_{\delta (t_{k})})<0, \end{array}$$

it can be got that

3.18 $$V_{\delta (t_{k})}(t_{k+1})-\lambda_{\;}^{-2(t_{k+1}-t_{k})}V_{\delta (t_{k})}(t_{k})<0.$$

According to Schur complement theorem, equation (3.17) is equivalent to

3.19 $$\left[\begin{array}{cc} -I_{N}⊗P_{\delta (t_{k})} & \left(I_{N}⊗P_{\delta (t_{k})}{\bar{A}}_{1}-\rho L⊗P_{\delta (t_{k})}{\bar{K}}_{2}C\right)\\ {}* & -\lambda_{\delta (t_{k})}^{-2(t_{k+1}-t_{k})}(I_{N}⊗P_{\delta (t_{k})}) \end{array}\right]<0.$$

It is noted that *L*^*T*^ = *L* in the MASs with undirected connected topology. Hence, there exists an orthogonal matrix *H* to satisfy $L=H^{T}\Lambda H$, where $\Lambda =\mathrm{diag}\{\psi_{1},\ldots ,\psi_{N}\}$ and *ψ*_*i*_ is the eigenvalue of *L*. The left multiplication and right multiplication of inequality (3.19) by $\left[\begin{array}{cc} H^{T}⊗I & O\\ O & H^{T}⊗I \end{array}\right]$ and $\left[\begin{array}{cc} H⊗I & O\\ O & H⊗I \end{array}\right]$, respectively. Equation (3.20) can be obtained from inequality (3.19) by making proper matrix transformation:

3.20 $$\Xi =\mathrm{diag}\{\Xi_{1},\ldots ,\Xi_{N}\}<0,$$

where $\Xi_{i}=\left[\begin{array}{cc} -P_{\delta (t_{k})} & P_{\delta (t_{k})}{\bar{A}}_{1}-\rho \psi_{i}P_{\delta (t_{k})}{\bar{K}}_{2}C\\ {}* & -\lambda_{\delta (t_{k})}^{-2(t_{k+1}-t_{k})}P_{\delta (t_{k})} \end{array}\right]$ (*i* = 1, 2, …, *N*). Obviously, inequality (3.18) holds if (3.11) holds. It means that $V_{\delta (t_{k})}(t_{k})$ decays exponentially along the respective subsystem trajectories. Further, the following equation (3.21) can be obtained:

3.21 $$\begin{array}{cc} & V_{\delta (t_{\iota })}(t_{k})\leq \lambda_{\chi_{\iota }}^{-2(t_{k}-t_{k_{\iota }})}V_{\delta (t_{\iota })}(t_{k_{\iota }})\\ \mathrm{and}\;\;\;\;\;\; & V_{\delta (t_{\varphi })}(t_{k_{\iota }})\leq \lambda_{\chi_{\varphi }}^{-2(t_{k_{\iota }}-t_{k_{\varphi }})}V_{\delta (t_{\varphi })}(t_{k_{\varphi }}). \end{array}\}$$

According to (3.12) and (3.15), it can be got that

3.22 $$\begin{array}{rl} V_{\chi_{\varphi }}(t_{k_{\varphi }}) & =e_{\chi_{\varphi }}^{T}(t_{k_{\varphi }})P_{\chi_{\varphi }}e_{\chi_{\varphi }}(t_{k_{\varphi }})\\ & =e_{\chi_{{\;}_{\varphi -1}}}^{T}(t_{k_{\varphi }})P_{\chi_{\varphi }}e_{\chi_{\varphi -1}}(t_{k_{\varphi }})\\ & \leq ue_{\chi_{{\;}_{\varphi -1}}}^{T}(t_{k_{\varphi }})P_{\chi_{\varphi -1}}e_{\chi_{\varphi -1}}(t_{k_{\varphi }})\\ & =uV_{\chi_{\varphi -1}}(t_{k_{\varphi }}). \end{array}$$

Using the definition of ADT, the following recursive equation can be obtained by combining (3.21) and (3.22):

3.23 $$\begin{array}{rl} V_{\delta (t_{k})}(t_{k}) & \leq \lambda_{\chi_{\iota }}^{-2(t_{k}-t_{k_{\iota }})}V_{\chi_{\iota }}(t_{k_{\iota }})\\ & \leq \mu \lambda_{\chi_{\iota }}^{-2(t_{k}-t_{k_{\iota }})}V_{\chi_{\iota -1}}(t_{k_{\iota }})\\ & \leq \mu \lambda_{\chi_{\iota }}^{-2(t_{k}-t_{k_{\iota }})}\lambda_{\chi_{\iota -1}}^{-2(t_{k_{\iota }}-t_{k_{\iota -1}})}V_{\chi_{\iota -1}}(t_{k_{\iota -1}})\\ \vdots \\ & \leq \mu^{N_{\delta }(t_{0},t_{k})}\lambda_{\chi_{\iota }}^{-2(t_{k}-t_{k_{\iota }})}\lambda_{\chi_{\iota -1}}^{-2(t_{k_{\iota }}-t_{k_{\iota -1}})}\cdots \lambda_{\chi_{0}}^{-2(t_{k_{1}}-t_{0})}V_{\delta (t_{0})}(t_{0})\\ & =\mu^{N_{\delta }(t_{0},t_{k})}\prod_{\varphi =1}^{d+1}\lambda_{\chi_{\varphi }}^{-2\tau_{\alpha }n_{\chi_{\varphi }}}V_{\delta (t_{0})}(t_{0})\\ & =\mu^{N_{\delta }(t_{0},t_{k})}\prod_{\varphi =1}^{d+1}\lambda_{\chi_{\varphi }}^{-2\tau_{\alpha }\gamma_{\chi_{\varphi }}N_{\delta }[t_{0},t_{k})}V_{\delta (t_{0})}(t_{0}). \end{array}$$

When inequality (3.13) is established, (3.23) can be rewritten as

3.24 $$V_{\delta (t_{k})}(t_{k})\leq \mu^{N_{\delta }(t_{0},t_{k})}\lambda^{-2\tau_{\alpha }N_{\delta }[t_{0},t_{k})}V_{\delta (t_{0})}(t_{0}).$$

Since $N_{\delta }[t_{0},t_{k})\leq (t_{k}-t_{0})/\tau_{a}$, it can be obtained from (3.24) that

3.25 $$V_{\delta (t_{k})}(t_{k})\leq \mu^{\left(-2(t_{k}-t_{0}))/(-2\tau_{\alpha }\right)}\lambda^{-2(t_{k}-t_{0})}V_{\chi_{0}}(t_{0}).$$

Let $\rho (\tau_{\alpha },\lambda )=\mu^{-(1/2\tau_{\alpha })}\lambda$, then (3.25) can be rewritten as

3.26 $$V_{\delta (t_{k})}(t_{k})\leq \rho^{-2(t_{k}-t_{0})}(\tau_{\alpha },\lambda )V_{\delta (t_{0})}(t_{0}).$$

Let $\psi_{1}=\min_{\delta (t_{k})\in Z_{0}}\lambda_{\min}(P_{\delta (t_{k})})$ and $\psi_{2}=\max_{\delta (t_{k})\in Z_{0}}\lambda_{\max}(P_{\delta (t_{k})})$, then

3.27 $$\;\psi_{1}∥e(t_{k}){∥}^{2}\leq V_{\delta (t_{k})}(t_{k})\leq \rho^{-2(t_{k}-t_{0})}(\tau_{\alpha },\lambda )V_{\delta (t_{0})}(t_{0})\leq \rho^{-2(t_{k}-t_{0})}(\tau_{\alpha },\lambda )\psi_{2}\|e(t_{0}){\|}^{2}.$$

From (3.27), it can be got that

3.28 $$\|e(t_{k})\|\leq \sqrt{\frac{\psi_{2}}{\psi_{1}}}\rho^{-(t_{k}-t_{0})}(\tau_{\alpha },\lambda )\|e(t_{0})\|.$$

Obviously, $\rho (\tau_{\alpha },\lambda )>1$ if (3.14) holds. This means that system (3.8) with *ω*(*t*_*k*_) = 0 is exponentially stable and satisfies the exponential decay rate $\rho (\tau_{\alpha },\lambda )$, which completes the proof. ▪

**Remark 3.5.**

Let ${\bar{\lambda }}_{s}=\min\{\lambda_{2},\lambda_{3},\ldots ,\lambda_{d+1}\}$, ${\bar{\lambda }}_{1}>\lambda$ and ${\bar{\lambda }}_{1}>{\bar{\lambda }}_{s}$. Since $\alpha =\sum_{\chi_{\varphi }=2}^{d+1}\gamma_{\chi_{\varphi }}$ and $\sum_{\chi_{\varphi }=1}^{d+1}\gamma_{\chi_{\varphi }}=1$, it can be got that $\prod_{\varphi =1}^{d+1}\lambda_{\chi_{\varphi }}^{\gamma_{\chi_{\varphi }}}\geq {\bar{\lambda }}_{1}^{\gamma_{\chi_{{\;}_{1}}}}\prod_{\varphi =2}^{d+1}\lambda_{\chi_{\varphi }}^{\gamma_{\chi_{\varphi }}}={\bar{\lambda }}_{1}^{1-\sum_{\chi_{\varphi }=2}^{d+1}\gamma_{\chi_{\varphi }}}{\bar{\lambda }}_{s}^{\sum_{\chi_{\varphi }=2}^{d+1}\gamma_{\chi_{\varphi }}}={\bar{\lambda }}_{1}^{\left(1-\alpha \right)}{\bar{\lambda }}_{s}^{\alpha }$. According to (3.13), we can get ${\bar{\lambda }}_{1}^{1-\alpha }{\bar{\lambda }}_{s}^{\alpha }>\lambda >1$, which means the following equation holds:

3.29 $$\alpha <\bar{\alpha }=\frac{\ln({\bar{\lambda }}_{1}/\lambda )}{\ln({\bar{\lambda }}_{1}/\lambda_{s})}.$$

Since $\lambda =\rho^{2}(\tau_{\alpha },\lambda )\mu^{1/\tau_{\alpha }}$, so it can be got that

3.30 $$\bar{\alpha }=\frac{\ln{\bar{\lambda }}_{1}\rho^{2}(\tau_{\alpha },\lambda )\mu^{-(1/\tau_{\alpha })}}{\ln({\bar{\lambda }}_{1}/\lambda_{s})}=-\beta_{1}\ln\rho (\tau_{\alpha },\lambda )+\beta_{2},$$

where $\beta_{1}=(2/\ln({\bar{\lambda }}_{1}/\lambda_{s}))$ and $\beta_{2}=(\ln{\bar{\lambda }}_{1}-(1/\tau_{\alpha })\ln\mu )/\ln({\bar{\lambda }}_{1}/\lambda_{s})$.

Equation (3.29) shows the upper limit of the DPL rate to ensure that system (3.8) meets exponential stability. And equation (3.30) shows that the upper bound of the DPL rate is a monotonic decreasing function of the exponential decay rate $\rho (\tau_{\alpha },\lambda )$, that is, the smaller the DPL rate, the greater the exponential decay rate of the system.

Next, *H*_∞_ performance conditions of system (3.8) to bounded disturbance *ω*(*t*_*k*_) ≠ 0 will be analysed.

**Theorem 3.6.**

*For MASs with undirected connected topology, if there exist scalar* *γ* > 0, *λ* > 1, *μ* ≥ 1, $\lambda_{\chi_{\varphi }}>1$, ($\chi_{\varphi }\in Z_{\delta },\varphi =1,\ldots ,d+1$), *positive definite matrix* $P_{\delta (t_{k})}$, $P_{\alpha }$ *and* $P_{\beta }$ ($\delta (t_{k}),\alpha ,\beta \in Z_{\delta }$) *to satisfy conditions of theorem 3.4 and the following inequalities:*

3.31 $$\Theta_{i}=\left[\begin{array}{ccc} -P_{\delta (t_{k})} & P_{\delta (t_{k})}{\bar{A}}_{1}-\rho \psi_{i}P_{\delta (t_{k})}{\bar{K}}_{2}C & P_{\delta (t_{k})}E\\ {}* & -\lambda_{\delta (t_{k})}^{-2(t_{k+1}-t_{k})}P_{\delta (t_{k})}+I & O\\ {}* & * & -\gamma^{2}I \end{array}\right]<0,\;i=1,2,\ldots ,N$$

*and*

3.32 $$1-\mu \lambda_{\chi_{\varphi }}^{-2(t_{k_{\varphi +1}}-t_{{\;}_{\varphi }})}>0,\varphi =1,\ldots ,d,$$

*then the error dynamic system* (3.8) *with* *ω*(*t*_*k*_) ≠ 0 *has* *H*_∞_ *performance, where* *γ* *is the performance level*.

**Proof.**

Let us choose the following cost function:

3.33 $$J=\sum_{k=0}^{\infty }[e^{T}(t_{k})e(t_{k})-\gamma^{2}\omega^{T}(t_{k})\omega (t_{k})].$$

According to definition 3.3, if *J* < 0 can be maintained, the above theorem can be proved. The same candidate Lyapunov function described as (3.15) is selected. When *ω*(*t*_*k*_) ≠ 0, combining with equation (3.8) and (3.15), it can be got that

3.34 $$\begin{array}{rl} & V_{\delta (t_{k})}(t_{k+1})-\lambda_{\delta (t_{k})}^{-2(t_{k+1}-t_{k})}V_{\delta (t_{k})}(t_{k})\\ & \;=e_{\;}^{T}(t_{k+1})P_{\delta (t_{k})}e(t_{k+1})-\lambda_{\;}^{-2(t_{k+1}-t_{k})}e_{\;}^{T}(t_{k})(I_{N}⊗P_{\delta (t_{k})})e_{\;}(t_{k})\\ & \;=e_{\;}^{T}(t_{k}){\left(I_{N}⊗{\bar{A}}_{1}-(\rho L⊗{\bar{K}}_{2}C)\right)}^{T}(I_{N}⊗P_{\delta (t_{k})})(I_{N}⊗{\bar{A}}_{1}-(\rho L⊗{\bar{K}}_{2}C))e(t_{k})\\ & \;+e_{\;}^{T}(t_{k}){\left(I_{N}⊗{\bar{A}}_{1}-(\rho L⊗{\bar{K}}_{2}C)\right)}^{T}(I_{N}⊗P_{\delta (t_{k})})I_{N}⊗E_{1}\omega (t_{k})\\ & \;+\omega^{T}(t_{k}){\left(I_{N}⊗E_{1}\right)}^{T}(I_{N}⊗P_{\delta (t_{k})})(I_{N}⊗{\bar{A}}_{1}-(\rho L⊗{\bar{K}}_{2}C))e(t_{k})\\ & \;+\omega^{T}(t_{k}){\left(I_{N}⊗E_{1}\right)}^{T}(I_{N}⊗P_{\delta (t_{k})})I_{N}⊗E_{1}\omega (t_{k})\\ & \;-\lambda_{\delta (t_{k})}^{-2(t_{k+1}-t_{k})}e_{\;}^{T}(t_{k})(I_{N}⊗P_{\delta (t_{k})})e(t_{k}). \end{array}$$

Defining augmented vectors $\theta (t_{k})={\left[e_{\;}^{T}(t_{k})\;\omega^{T}(t_{k})\right]}^{T}$, (3.34) can be rewritten as

3.35 $$\begin{array}{rl} & V_{\delta (t_{k})}(t_{k+1})-\lambda_{\delta (t_{k})}^{-2(t_{k+1}-t_{k})}V_{\delta (t_{k})}(t_{k})=\theta^{T}(t_{k})\Phi_{\delta (t_{k})}\theta^{T}(t_{k})+\gamma^{2}\omega^{T}(t_{k})\omega (t_{k})-e^{T}(t_{k})e(t_{k}), \end{array}$$

where

$$\begin{array}{rl} \Phi_{\delta (t_{k})} & =\left[\begin{array}{cc} -\lambda_{\delta (t_{k})}^{-2(t_{k+1}-t_{k})}(I_{N}⊗P_{\delta (t_{k})})P_{\delta (t_{k})}+I & O\\ O & -\gamma^{2}I \end{array}\right]\\ & \;+\left[\begin{array}{c} {\left(I_{N}⊗{\bar{A}}_{1}-(\rho L⊗{\bar{K}}_{2}C)\right)}^{T}(I_{N}⊗P_{\delta (t_{k})})\\ {\left(I_{N}⊗E\right)}^{T}(I_{N}⊗P_{\delta (t_{k})}) \end{array}\right](I_{N}⊗P_{\delta (t_{k})}^{-1})\\ & \;[(I_{N}⊗P_{\delta (t_{k})})(I_{N}⊗{\bar{A}}_{1}-(\rho L⊗{\bar{K}}_{2}C))\;(I_{N}⊗P_{\delta (t_{k})})I_{N}⊗E_{1}]. \end{array}$$

According to Schur complement theorem, when

3.36 $$\left[\begin{array}{ccc} -I_{N}⊗P_{\delta (t_{k})} & \left(I_{N}⊗P_{\delta (t_{k})})(I_{N}⊗{\bar{A}}_{1}-(\rho L⊗{\bar{K}}_{2}C)\right) & (I_{N}⊗P_{\delta (t_{k})})I_{N}⊗E_{1}\\ {}* & -\lambda_{\delta (t_{k})}^{-2(t_{k+1}-t_{k})}(I_{N}⊗P_{\delta (t_{k})})+I & O\\ {}* & * & -\gamma^{2}I \end{array}\right]<0$$

holds, it can be got that $\theta (t_{k})\Phi_{\delta (t_{k})}\theta^{T}(t_{k})<0$. Then we can get

3.37 $$e^{T}(t_{k})e(t_{k})-\gamma^{2}\omega^{T}(t_{k})\omega (t_{k})<\lambda_{\delta (t_{k})}^{-2(t_{k+1}-t_{k})}V_{\delta (t_{k})}(t_{k})-V_{\delta (t_{k})}(t_{k+1}).$$

Left and right multiplication matrices $\left[\begin{array}{ccc} H^{T}⊗I & O & O\\ {}* & H^{T}⊗I & O\\ {}* & * & H^{T}⊗I \end{array}\right]$ and $\left[\begin{array}{ccc} H⊗I & O & O\\ {}* & H⊗I & O\\ {}* & * & H⊗I \end{array}\right]$ of inequality (3.36), respectively, then (3.36) is equivalent to

$$\Theta =\mathrm{diag}\{\Theta_{1},\ldots ,\Theta_{N}\}<0,$$

where $\Theta_{i}=\left[\begin{array}{ccc} -P_{\delta (t_{k})} & P_{\delta (t_{k})}{\bar{A}}_{1}-\rho \psi_{i}P_{\delta (t_{k})}({\bar{K}}_{2}C) & P_{\delta (t_{k})}E\\ {}* & -\lambda_{\delta (t_{k})}^{-2(t_{k+1}-t_{k})}P_{\delta (t_{k})}+I & O\\ {}* & * & -\gamma^{2}I \end{array}\right]$, *i* = 1, 2, …, *N*. Obviously, (3.36) holds if (3.31) holds. Further, the following equation can be obtained recursively from inequality (3.37):

$$\begin{array}{rl} & e^{T}(t_{k_{d}})e(t_{k_{d}})-\gamma^{2}\omega^{T}(t_{k_{d}})\omega (t_{k_{d}})<\lambda_{\chi_{d}}^{-2(t_{k}-t_{k_{d}})}V_{\chi_{d}}(t_{k_{d}})-V_{\chi_{d}}(t_{k})\\ & e^{T}(t_{k_{d-1}})e(t_{k_{d-1}})-\gamma^{2}\omega^{T}(t_{k_{d-1}})\omega (t_{k_{d-1}})<\lambda_{\chi_{d-1}}^{-2(t_{k_{d}}-t_{k_{d-1}})}V_{\chi_{d-1}}(t_{k_{d-1}})-V_{\chi_{d-1}}(t_{k_{d}})\\ \vdots \\ & e^{T}(t_{0})e(t_{0})-\gamma^{2}\omega^{T}(t_{k_{0}})\omega (t_{k_{0}})<\lambda_{\chi_{0}}^{-2(t_{k_{1}}-t_{k_{0}})}V_{\chi_{0}}(t_{0})-V_{\chi_{0}}(t_{k_{1}}). \end{array}$$

Adding the two sides of the above inequality separately gives

3.38 $$\begin{array}{rl} & \sum_{t=t_{0}}^{t_{k}}e^{T}(t)e(t)-\sum_{t=t_{0}}^{t_{k}}\gamma^{2}\omega^{T}(t)\omega (t)<\lambda_{\chi_{0}}^{-2(t_{k_{1}}-t_{{\;}_{0}})}V_{\chi_{0}}(t_{0})-(1-\mu \lambda_{\chi_{1}}^{-2(t_{k_{2}}-t_{{\;}_{k_{1}}})})V_{\chi_{0}}(t_{k_{1}})\\ & \;-(1-\mu \lambda_{\chi_{1}}^{-2(t_{k_{3}}-t_{k_{2}})})V_{\chi_{1}}(t_{k_{2}})-\cdots -V_{\sigma (t_{k})}(t_{k}). \end{array}$$

If (3.32) holds, it can be obtained from (3.38) that

3.39 $$\sum_{t=t_{0}}^{t_{k}}e^{T}(t)e(t)-\sum_{t=t_{0}}^{t_{k}}\gamma^{2}\omega^{T}(t)\omega (t)<\lambda_{\chi_{0}}^{-2(t_{k_{1}}-t_{{\;}_{0}})}V_{\chi_{0}}(t_{0}),$$

where *t*_0_ denotes the initial moment of the system. Let *t*_0_ = 0, then it can be obtained from equation (3.39) under zero initial conditions that

3.40 $$\sum_{t=0}^{t_{k}}e^{T}(t)e(t)<\sum_{t=0}^{t_{k}}\gamma^{2}\omega^{T}(t)\omega (t).$$

Consequently, it can be got that $\sum_{t=0}^{\infty }e^{T}(t)e(t)<\sum_{t=0}^{\infty }\gamma^{2}\omega^{T}(t)\omega (t)$ when *t*_*k*_ → ∞, i.e. *J* < 0, which completes the proof. ▪

## 4. Simulation

**Figure 3.**
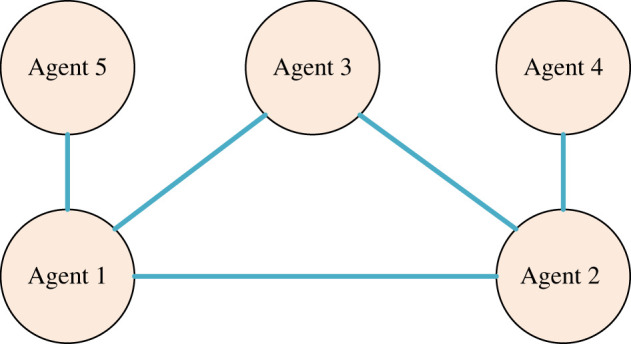
Undirected topology of MASs.

In this section, some numerical simulations are given to verify the results proposed in the paper. MASs with five nodes and undirected topology are given in figure 3. $A=\left[\begin{array}{ccccc} 0 & 1 & 1 & 0 & 1\\ 1 & 0 & 1 & 1 & 0\\ 1 & 1 & 0 & 0 & 0\\ 0 & 1 & 0 & 0 & 0\\ 1 & 0 & 0 & 0 & 0 \end{array}\right]$.

Parameter matrices of agent subsystem are chosen as $A_{0}=\left[\begin{array}{cc} -0.8 & -0.01\\ 1 & 0.1 \end{array}\right]$, $B_{0}=\left[\begin{array}{c} 0.4\\ 0.1 \end{array}\right]$. The sampling period of the system is *h* = 0.1. By discretizing the system with a fixed sampling period *h*, the parameter matrix without DPL can be obtained as $A_{\chi_{1}}=\left[\begin{array}{cc} 0.9920 & -0.0001\\ 0.0100 & 1.0010 \end{array}\right]$, $B_{\chi_{1}}=\left[\begin{array}{c} 0.0040\\ 0.0010 \end{array}\right]$. Assuming the maximum number of continuous DPL of the system *d* = 3, then the switching subsystem parameters can be obtained by discretion with a variable sampling period, which are shown as $A_{\chi_{2}}=\left[\begin{array}{cc} 0.9841 & 0.0002\\ 0.0199 & 0.0020 \end{array}\right]$, $B_{\chi_{2}}=\left[\begin{array}{c} 0.0079\\ 0.0021 \end{array}\right]$, $A_{\chi_{3}}=\left[\begin{array}{cc} 0.9763 & 0.0003\\ 0.0297 & 0.0030 \end{array}\right]$, $B_{\chi_{3}}=\left[\begin{array}{c} 0.0119\\ 0.0032 \end{array}\right]$, $A_{\chi_{4}}=\left[\begin{array}{cc} 0.9685 & 0.0004\\ 0.0394 & 0.0040 \end{array}\right]$, $B_{\chi_{4}}=\left[\begin{array}{c} 0.0157\\ 0.0043 \end{array}\right]$.

Assume that at [0, 50*h*], the numbers of activations of subsystems are $n_{\chi_{1}}=44,n_{\chi_{2}}=2,n_{\chi_{3}}=2$ and $n_{\chi_{4}}=2$. The switching sequence is $\overset{6}{\overbrace{s_{\chi_{1}}\cdots s_{\chi_{1}}}}s_{\chi_{2}}\overset{6}{\overbrace{s_{\chi_{1}}\cdots s_{\chi_{1}}}}s_{\chi_{3}}\overset{6}{\overbrace{s_{\chi_{1}}\cdots s_{\chi_{1}}}}s_{\chi_{4}}$ $\overset{6}{\overbrace{s_{\chi_{1}}\cdots s_{\chi_{1}}}}s_{\chi_{4}}\overset{6}{\overbrace{s_{\chi_{1}}\cdots s_{\chi_{1}}}}s_{\chi_{3}}\overset{6}{\overbrace{s_{\chi_{1}}\cdots s_{\chi_{1}}}}s_{\chi_{2}}\overset{6}{\overbrace{s_{\chi_{1}}\cdots s_{\chi_{1}}}}$. It can be got that $\tau_{\alpha }=0.42$, *χ*_0_ = 0.88, $\gamma_{\chi_{1}}=\gamma_{\chi_{2}}=\gamma_{\chi_{3}}=0.04$ and *α* = 0.12. Let $\lambda_{\chi_{1}}=1.44$, $\lambda_{\chi_{2}}=\lambda_{\chi_{3}}=1.25$, $\lambda_{\chi_{4}}=1.16$, *λ* = 1.4, *μ* = 1.07, ${\bar{\lambda }}_{1}=1.44$, *λ*_*s*_ = 1.16, *ρ* = 0.5, ϵ=3, *γ* = 0.7, $F=\left[\begin{array}{c} 0\\ 1 \end{array}\right]$ and $E=\left[\begin{array}{c} c1\\ 1 \end{array}\right]$. Combining the inequalities in theorems 3.4 and 3.6, it can be got that ${\bar{K}}_{2}=\left[\begin{array}{cc} 0.0078 & 0.0101\\ 0.005 & 0.0466\\ 0.0073 & 0.0571 \end{array}\right]$. The disturbance signal of the system is taken as $d_{i}(t_{k})=\sin(2\pi t_{k})\;*\;\eta (t_{k})$, where *η*(*t*_*k*_) denotes the Gaussian white noise signal with amplitude of 0.1.

**Figure 4.**
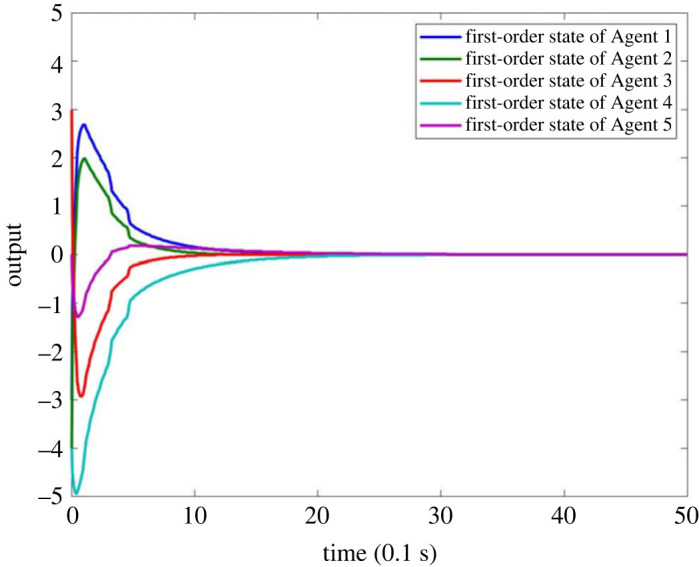
First-order state output of MASs without fault.

**Figure 5.**
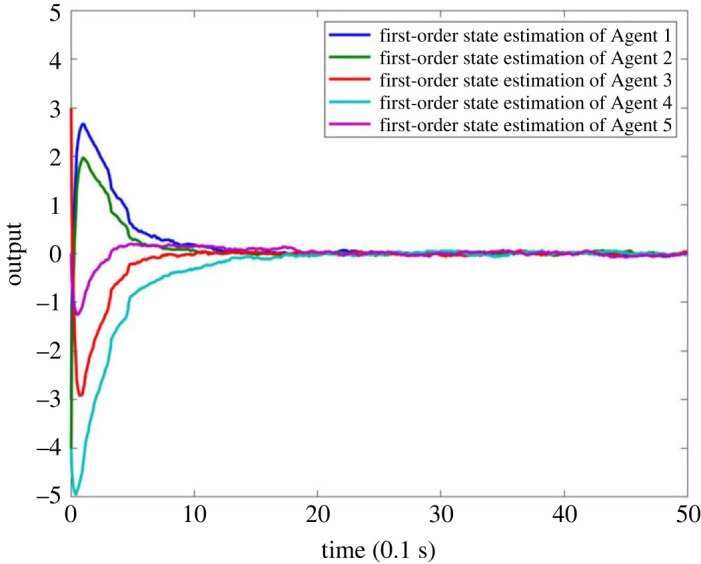
Estimation output of first-order state of MASs without fault.

**Figure 6.**
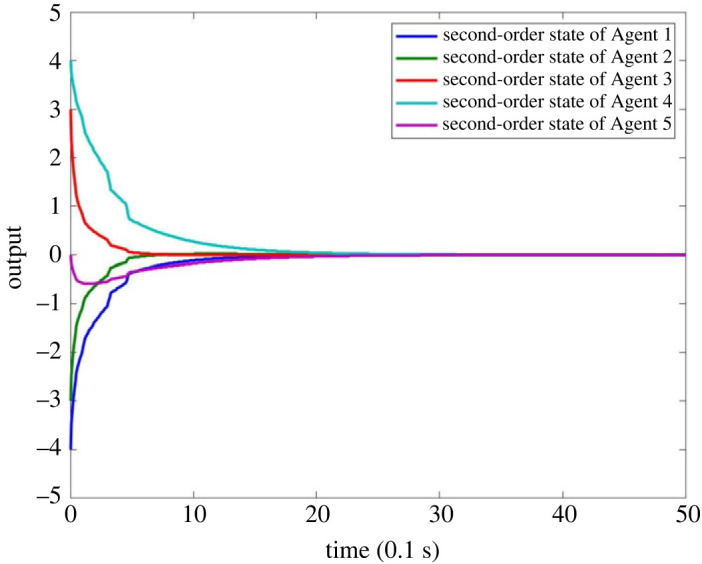
Second-order state output of MASs without fault.

**Figure 7.**
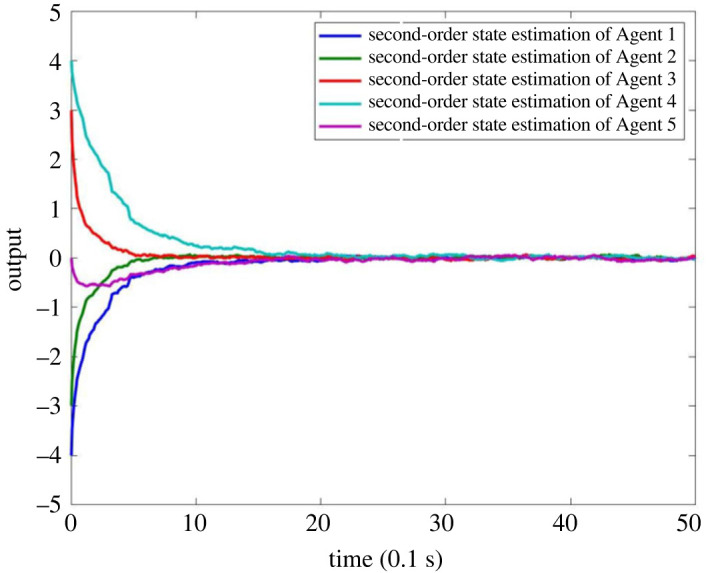
Estimation output of second-order state of MASs without fault.

**Figure 8.**
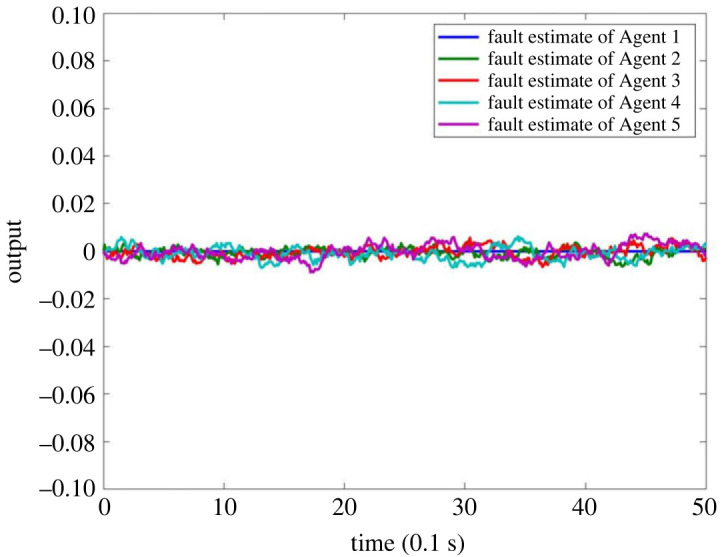
FE output of MASs without fault.

When there is no fault in MASs, the result is as shown in figures 4–8. Figures 4 and 6 show the actual state output of MASs, figures 5 and 7 the estimation outputs of MASs. It can be seen that the observer can effectively estimate the system state when there is no fault. Figure 8 is the FE output. When there is no fault, the outputs are irregular curves near zero, which show that the observer can effectively estimate the fault signal when there is no fault.

**Figure 9.**
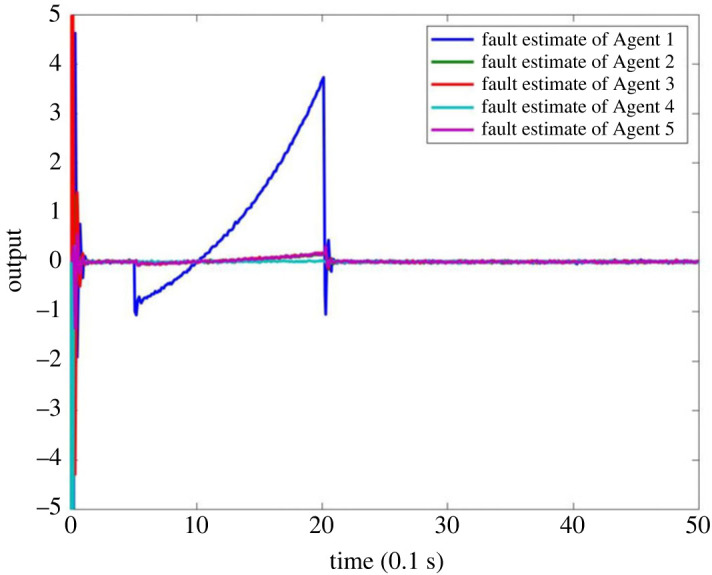
FE output of MASs with single fault.

**Figure 10.**
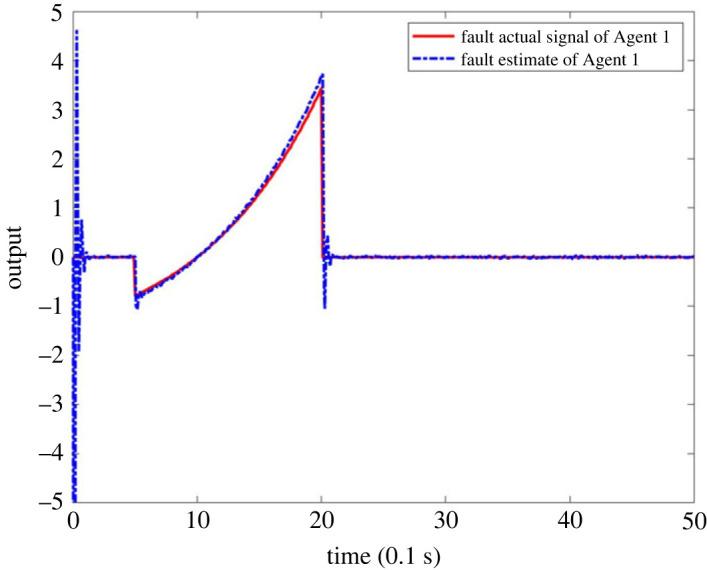
Actual fault signal and FE output of MASs with single fault.

When there is a signal fault in MASs, suppose the fault occurs on Agent 1, and the fault signal is described as $\;f_{1}(t_{k})=2(e^{\left(t_{k}-1\right)}-1)$ when 0.5 ≤ *t*_*k*_ ≤ 2 and *f*_1_(*t*_*k*_) = 0 when *t*_*k*_ < 0.5 or *t*_*k*_ > 2. The outputs of system are shown in figures 9 and 10. It can be seen that the observer can make a timely response to the failure of node 1 in figure 9. Since the fault only occurs in one agent, the observer’s output to other nodes is approximately zero, which also shows that the observer can carry out the fault alarm for signal fault. Figure 10 is the output of observer’s comparison between the estimated value of the fault and actual value. It can be seen that the estimator can accurately estimate the fault signal, which also verifies the effectiveness of FE for a single fault of the system.

**Figure 11.**
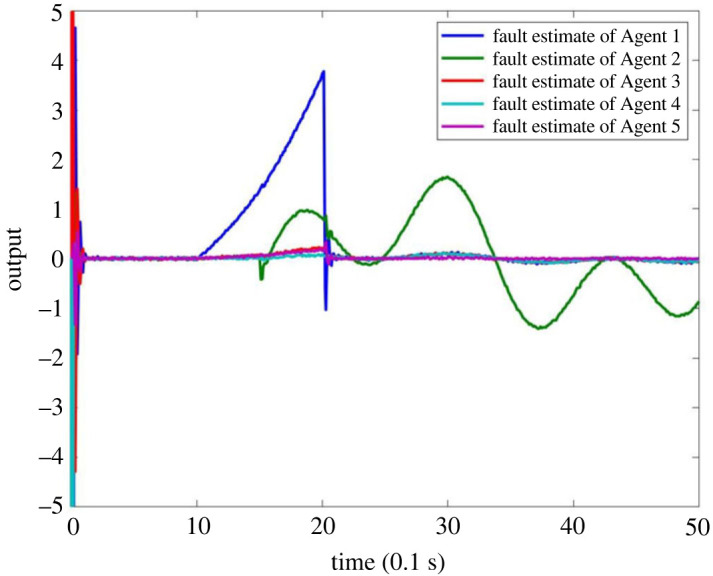
FE output of MASs with multiple faults.

**Figure 12.**
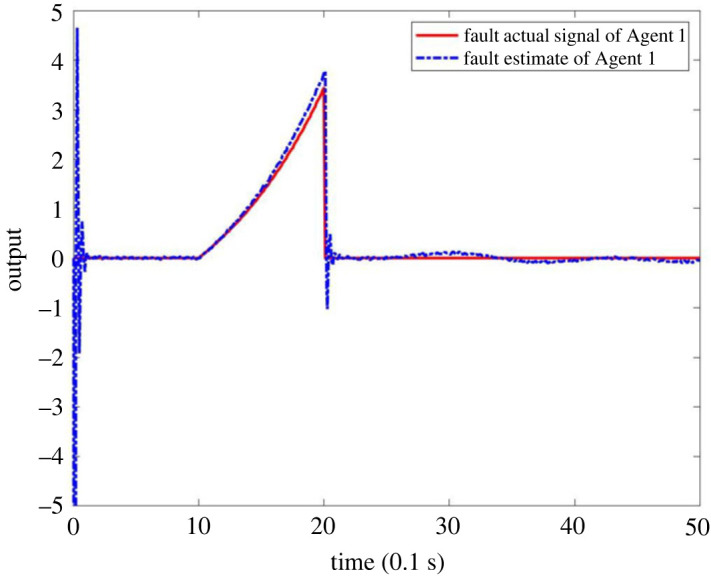
Actual fault signal and FE output of Agent 1.

**Figure 13.**
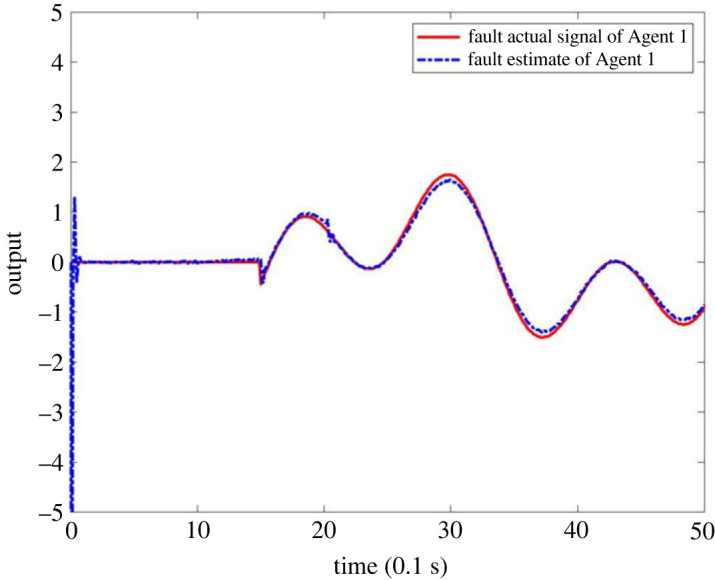
Actual fault signal and FE output of Agent 2.

When multiple faults occur in the system MASs, it is assumed that the fault occurs on Agent 1 and Agent 2, respectively, and the fault signal is described as follows: $\;f_{1}(t_{k})=2(e^{\left(t_{k}-1\right)}-1)$ when 1 ≤ *t*_*k*_ ≤ 2 and *f*_1_(*t*_*k*_) = 0 when *t*_*k*_ < 1 or *t*_*k*_ > 2, $\;f_{2}(t_{k})=\sin(0.5t_{k}-1)+\cos(0.2t_{k}-1)$ when *t*_*k*_ ≥ 1.5 and *f*_2_(*t*_*k*_) = 0 when *t*_*k*_ < 1.5. The output results are as shown in figures 11–13. Figure 11 shows that the observers can make a timely response to Agent 1 and Agent 2. Figures 12 and 13 shows that the estimator can effectively estimate the faults that occur in Agent 1 and Agent 2. This shows that the observer is effective in FE for multiple faults.

## 5. Conclusion

In this paper, the problem of distributed robust FE for MASs with DPL is investigated. Firstly, by decomposing and transforming the node’s information dynamic model, the problem of DPL is transformed into the problem of time-varying sampling period, and based on this, MAS switching dynamic models with time-varying sampling period are constructed. Secondly, intermediate observers independent of the matching relationship of the system parameter matrix are constructed, which can estimate the system state and multiple fault signals simultaneously. Thirdly, the output bias dynamics model of the observer is constructed, and the design problem of the observer is converted into the convergence problem of the output error dynamics. It is shown that the error dynamics satisfy exponential stability and have *H*_∞_ performance to rebounded disturbance. It is worth noting that this paper only considers the FE for undirected MASs with communication constraints. Subsequent research will further develop a robust fault estimation for directed MASs with communication constraints.

## Data Availability

This article has no additional data.

## References

[1] Gianvito D, Maria proof, Agostino MM. 2022 Maximizing convergence speed for second order consensus in leaderless multi-agent systems. IEEE/CAA J. Autom. Sin. **9**, 259-269. (10.1109/JAS.2021.1004320)

[2] Huang X, Chen Y, Zhan J. 2022 Observer-based consensus control for multi-agent systems with measurement noises and external disturbances. Int. J. Robust Nonlinear Control **1**, 344-357. (10.1002/rnc.5821)

[3] Yang Y, Xu D, Ma T, Su X. 2021 Adaptive cooperative terminal sliding mode control for distributed energy storage systems. IEEE Trans. Circuits Syst. **68**, 434-443. (10.1109/TCSI.2020.3027376)

[4] Liu Y, Li Z, Jiang Z, He Y. 2022 Prospects for multi-agent collaboration and gaming: challenge, technology, and application. Front. Inform. Technol. Electron. Eng. **23**, 1002-1009. (10.1631/FITEE.2200055)

[5] Dang TB, Tran MH, Le DT, Zalyubovskiy VV, Ahn H, Choo H. 2018 Trend-adaptive multi-scale PCA for data fault detection in IoT networks. In *2018 Int. Conf. on Information Networking, Chiang Mai, Thailand, 10–12 January 2018*, pp. 744–749. (10.1109/ICOIN.2018.8343217)

[6] Peng Y, Qiao W, Qu L, Wang J. 2017 Sensor fault detection and isolation for a wireless sensor network-based remote wind turbine condition monitoring system. IEEE Trans. Ind. Appl. **54**, 1072-1079. (10.1109/TIA.2017.2777925)

[7] Shames I, Teixeira AM, Sandberg H, Johansson KH. 2011 Distributed fault detection for interconnected second order systems. Automatica **47**, 2757-2764. (10.1016/j.automatica.2011.09.011)

[8] Liu X, Gao X, Han J. 2016 Observer-based fault detection for high-order nonlinear multi-agent systems. J. Frankl. Inst. **1**, 72-94. (10.1016/j.jfranklin.2015.09.022) 26724972

[9] Chen X, Zhang K, Jiang B. 2018 Finite-time unknown input observer-based distributed fault diagnosis for multi agent systems with disturbances. Circuits Syst. Signal Process. **37**, 4215-4233. (10.1007/s00034-018-0764-1)

[10] Liang D, Yang Y, Li R. 2021 Finite-frequency *H*_−_/*H*_∞_ unknown input observer-based distributed fault detection for multi-agent systems. J. Frankl. Inst. **6**, 3258-3275. (10.1016/j.jfranklin.2021.01.042)

[11] Zhou D, Qin L, He X, Yan R, Deng R. 2018 Distributed sensor fault diagnosis for a formation system with unknown constant time delays. Sci. China Inf. Sci. **61**, 128-144. (10.1007/s11432-017-9309-3)

[12] Zhu J W, Yang G H, Wang H, Wang FL. 2016 Fault estimation for a class of nonlinear systems based on intermediate estimator. IEEE Trans. Autom. Control **61**, 2518-2524. (10.1109/TAC.2015.2491898)

[13] Zhu J, Yang G. 2018 Robust distributed fault estimation for a network of dynamical systems. IEEE Trans. Control Netw. Syst. **5**, 14-22. (10.1109/TCNS.2016.2567223)

[14] Zhu J, Yang G, Zhang W, Yu L. 2018 Cooperative fault tolerant tracking control for multiagent systems: an intermediate estimator-based approach. IEEE Trans. Cybern. **48**, 2972-2980. (10.1109/TCYB.2017.2753383) 29053463

[15] Han J, Liu XH, Gao XW, Wei XJ. 2019 Intermediate observer based robust distributed fault estimation for nonlinear multi-agent systems with directed graphs. IEEE Trans. Ind. Inf. **16**, 7426-7436. (10.1109/TII.2019.2958988)

[16] Liu XH, Han J, Wei XJ. 2020 Intermediate observer based distributed fault estimation for multi-agent systems. ACTA Autom. Sin. **46**, 142-152.

[17] Xing ML, Deng FQ, Hu ZP. 2020 Sampled-data consensus for multiagent systems with time delays and packet losses. IEEE Trans. Syst. Man Cybern.: Syst. **50**, 203-210. (10.1109/TSMC.2018.2815616)

[18] Ni JK, Shi P, Zhao Y, Wu ZH. 2021 Fixed-time output consensus tracking for high-order multi-agent systems with directed network topology and packet dropout. IEEE/CAA J. Autom. Sin. **8**, 817-836. (10.1109/JAS.2021.1003916)

[19] Li J, Wang J, Su Q, Wu CY, Zhao XQ. 2020 Fault detection for interconnected systems subject to packet dropouts via switching scheme. Int. J. Control Autom. Syst. **18**, 3031-3042. (10.1007/s12555-019-0358-0)

[20] Zhang DJ, Zhang YS. 2021 Fault detection for delta operator systems with uncertain packet dropout rate and time-varying sampling periods. Control Decis. **36**, 1101-1109.

[21] Zhang L, Gao H. 2010 Asynchronously switched control of switched linear systems with average dwell time. Automatica **46**, 953-958. (10.1016/j.automatica.2010.02.021)

[22] Yu L, Zhang WA. 2012 Design and analysis of networked measurement and control system: switching system processing method. Beijing, China: Science Press.

